# Proteomic analysis of lamellar bodies isolated from rat lungs

**DOI:** 10.1186/1471-2121-9-34

**Published:** 2008-06-24

**Authors:** Pengcheng Wang, Narendranath Reddy Chintagari, Jeyaparthasarathy Narayanaperumal, Sahlu Ayalew, Steven Hartson, Lin Liu

**Affiliations:** 1 Department of Physiological Sciences, Oklahoma State University, Stillwater, OK 74078, USA; 2 Department of Pathobiology, Oklahoma State University, Stillwater, OK 74078, USA; 3 Department of Biochemistry & Molecular Biology, Oklahoma State University, Stillwater, OK 74078, USA

## Abstract

**Background:**

Lamellar bodies are lysosome-related secretory granules and store lung surfactant in alveolar type II cells. To better understand the mechanisms of surfactant secretion, we carried out proteomic analyses of lamellar bodies isolated from rat lungs.

**Results:**

With peptide mass fingerprinting by Matrix Assisted Laser Desorption/Ionization – Time of Flight mass spectrometry, 44 proteins were identified with high confidence. These proteins fell into diverse functional categories: surfactant-related, membrane trafficking, calcium binding, signal transduction, cell structure, ion channels, protein processing and miscellaneous. Selected proteins were verified by Western blot and immunohistochemistry.

**Conclusion:**

This proteomic profiling of lamellar bodies provides a basis for further investigations of functional roles of the identified proteins in lamellar body biogenesis and surfactant secretion.

## Background

Lung surfactant is a surface active material. It reduces the surface tension of the air-liquid interface in alveoli, thus preventing alveoli from collapsing. Deficiency of surfactant at the alveolar surface results in respiratory distress syndromes (RDS) in both newborns and adults [1]. Lung surfactant is synthesized and secreted by alveolar type II cells. It is mainly composed of phospholipids and surfactant proteins A, B and C (SP-A, SP-B and SP-C). Major components of surfactant are synthesized in the endoplasmic reticulum and stored in specialized organelles called lamellar bodies [2].

Lamellar bodies are lysosome-related, large secretory organelles that are 1 to 2 micrometers in size [3]. Similar to lysosomes, lamellar bodies contain soluble lysosomal enzymes, such as acid phosphatase and lysosome associated membrane proteins [3]. Lamellar bodies have an acidic interior with a pH of about 6.1 or below [4]. However, lamellar bodies are different from lysosomes in that they are specialized for storage and secretion of surfactant rather than for degradation processes. The principal components of lamellar bodies, phospholipids, are tightly packed as concentric arrangements of bi-layer membranes. Secretion of surfactant involves the translocation, docking and fusion of lamellar bodies with the apical plasma membrane [5,6].

The molecular mechanisms that control the exocytosis of lamellar bodies are still poorly understood [5]. The recent emergence of powerful proteomic techniques has made it possible to profile the protein components in a specific tissue or subcellular organelle [7-9]. To better understand the regulation of lamellar body biogenesis and exocytosis, we performed proteomic analysis of lamellar bodies isolated from rat lungs. We carried out both one-dimensional and two-dimensional gel electrophoresis, followed by Matrix Assisted Laser Desorption/Ionization – Time Of Flight mass spectrometry (MALDI-TOF) and immunohistochemistry. Here, we report the first proteomic profiling of lamellar bodies, which will aid in defining the mechanisms of lamellar body exocytosis.

## Results

The method used to isolate lamellar bodies was based on the work of Chander et al. [10] and is in routine use in our laboratory [11-13]. The isolated lamellar body fraction consists of intact lamellar bodies and large amounts of concentric multilamellated membrane structures and contains no other organelles except for very low amounts of microsomes.

**Figure 1:**
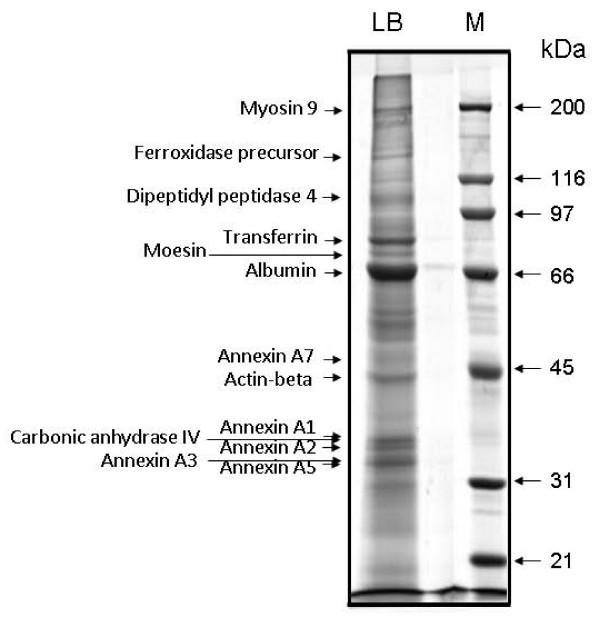
**One-dimensional SDS-PAGE of lamellar body proteins**. Lamellar bodies were isolated from perfused rat lungs as described in materials and methods. Approximately 100 μg of total protein were loaded on 10% Bis-Tris polyacrylamide gels. Protein bands were visualized by staining with Coomassie Brilliant Blue G-250.

**Figure 2:**
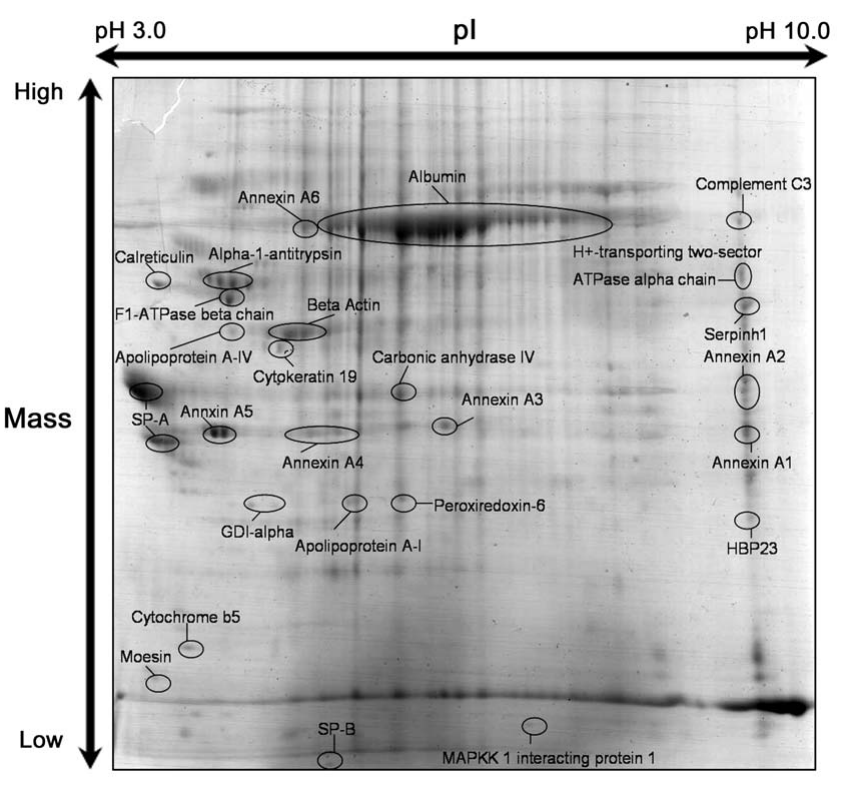
**Two-dimensional SDS-PAGE of lamellar body proteins**. Isolated lamellar bodies (500 μg of protein) were analyzed by 2-D polyacrylamide gel electrophoresis. Protein spots were visualized by staining with Coomassie Brilliant Blue G-250. Isoelectric points (pI) and molecular masses are shown on the top and the left of the panel, respectively.

One-dimensional sodium dodecyl sulfate polyacrylamide gel electrophoresis (SDS-PAGE) staining with colloidal Coomassie Brilliant Blue revealed more than 50 protein bands. Due to the complexity of protein components of lamellar bodies, we also performed two-dimensional PAGE to get better separation of the proteins. The two-dimensional gel electophoresis revealed more than 100 spots. Well-resolved protein bands or spots were harvested for peptide mass fingerprint analysis. Figs. 1 and 2 show some of the identified proteins from representive gels.

To identify protein components in lamellar bodies, we utilized trypsinolytic fingerprinting, MALDI-TOF mass spectrometry and statistically scored database searching (Mascot^®^). Table 1 lists the 44 proteins identified from the highly purified lamellar bodies. Listed for each protein include the National Center for Biotechnology Information (NCBI) accession number, the number of peptides matched and percent of sequence covered, the Mascot^® ^Probability Based Mowse (PBM) score and the molecular mass. By applying each of these proteomics search criteria, the proteins were identified with great confidence.

**Table 1:** Proteins identified in lamellar bodies with MALDI-TOF MS

Protein identified	NCBI accession #	Peptide matched	Sequence covered %	Mascot Score	Molecular mass
**Ca^2+^-binding proteins**					
Annexin A1	P07150	16	42	137	38674
Annexin A2	Q07936	16	49	129	38523
Annexin A3	LURT3	15	45	148	36341
Annexin A4	Q5U362	14	42	140	35871
Annexin A5	P14668	18	52	156	35591
Annexin A6	P48037	14	20	66	75575
Annexin A7	Q6IRJ7	8	17	57	49987
Calreticulin precursor	AAH62395	12	24	119	47966
					
**Structural proteins**					
Actin-beta	ATRTC	10	31	97	41724
Vimentin	P31000	20	38	119	53569
Myosin-9 (Nonmuscle myosin heavy chain IIa)	Q62812	47	24	143	226066
Tropomyosin 1	AAA42289	10	32	60	32938
Moesin (Membrane-organizing extension spike protein)	O35763	16	20	64	67566
Cytokeratin 19	AAR36876	15	38	108	44609
					
**Surfactant-related proteins**					
Surfactant protein A precursor	P08427	10	43	63	26272
Surfactant protein B	Q6IN44	7	19	62	41590
ABCA3	AAH88202	7		160	240735
Peroxiredoxin-6	NP_446028	8	33	78	24672
Apolipoprotein A-I precursor	AAH89820	10	35	84	30043
Apolipoprotein A-IV	AAH91159	16	39	122	44429
Lysosome membrane protein II	P27615	8	16	52	53925
					
**Ion channels**					
H^+^-transporting two-sector ATPase alpha chain precursor	J05266	12	29	57	58790
F1-ATPase beta chain	1MABB	10	32	83	49043
Unnamed protein product	CAF05438	20	14	113	146309
					
**Membrane traffic**					
EHD1	AAH82030	11	20	65	60565
					
**Protein processing**					
Serpinh1 protein	Q5RJR9	7	18	56	46532
Dipeptidyl peptidase IV membrane-bound form precursor	A39914	15	20	85	90869
Alpha-1-antitrypsin precursor	AAA40788	10	24	87	45807
					
**Signal transduction**					
Mitogen-activated protein kinase kinase 1 interacting protein 1	AAH86353	6	57	65	13571
Rho GDP dissociation inhibitor (GDI) alpha	Q5XI73	7	36	90	23393
Rho-associated kinase beta	AAB37571	24	18	71	159527
					
**Miscellaneous proteins**					
Albumin precursor	P02770	17	30	128	68674
Poly-ubiquitin	Q63654	5	44	82	11234
Complement C3 precursor	625256	13	7	61	186342
Heme-binding 23 K protein (HBP23)	1QQ2A	7	28	58	22095
Putative alpha (1,3) fucosyltransferase	CAC81972	10	19	59	42931
Periaxin	I58157	19	13	102	146948
Carbonic anhydrase IV	AAH97329	10	25	72	35054
Heat shock 70 kDa protein 5	AAH62017	19	33	95	72302
Protein disulfide isomerase associated 3	AAH62393	16	32	95	56588
Transferrin	AAP97736	14	19	80	76346
Ferroxidase precursor	A35210	11	9	122	120588
RSB-11-77 protein	NP_872610	12	28	75	42058
Cytochrome b5	AAB67609	13	27	78	11400

**Figure 3:**
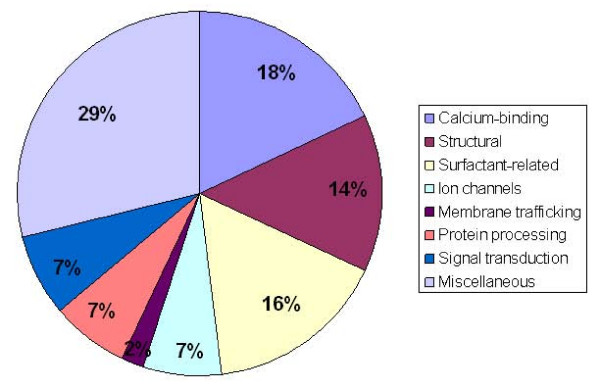
**Pie chart showing the functional classifications**. The 44 identified proteins were categorized into 8 different functional categories. The pie chart represents the percentage of identified proteins under each category. The percentages are shown within the pie chart.

The functional classification was performed by a literature search in the Pubmed database. The functional categories include calcium-binding proteins, structural proteins, surfactant-related proteins, ion channels, membrane traffic, protein processing, signal transduction and miscellaneous proteins (Fig. 3).

**Figure 4:**
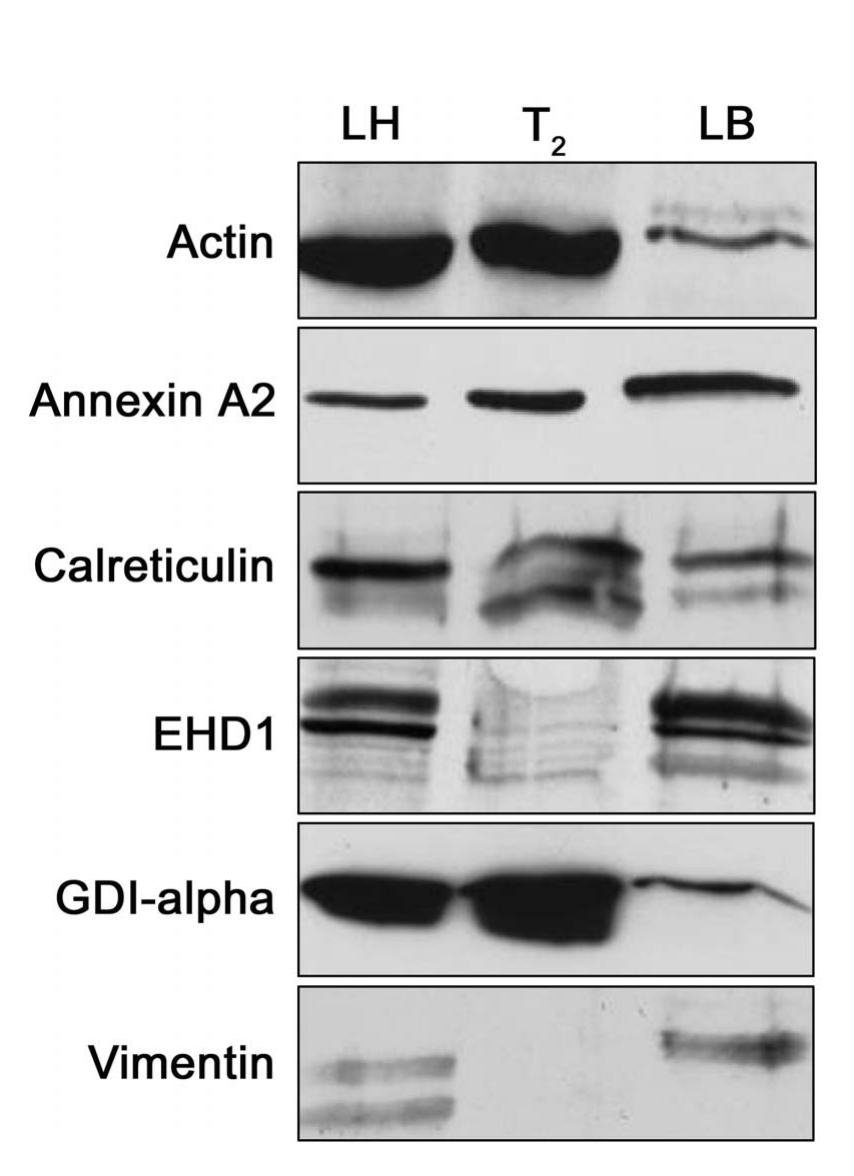
**Western blotting of selected proteins identified in lamellar bodies**. Lung tissue homogenate (LH), freshly isolated type II cells (T_2_), and lamellar body fraction (LB) were lysed and 20 μg of total protein were separated by 10% SDS-PAGE and probed with corresponding antibodies.

**Figure 5:**
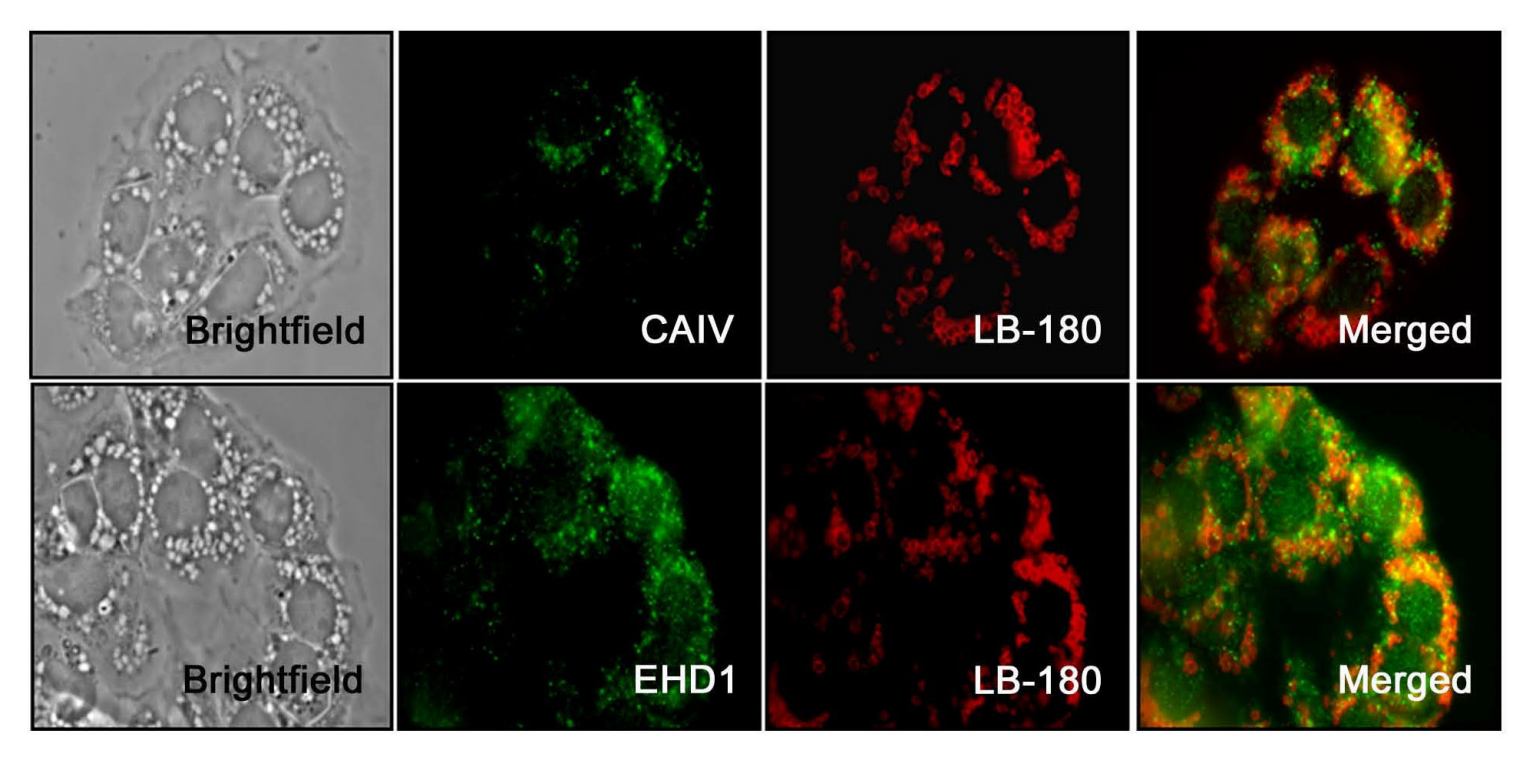
**Immunolocalization of selected proteins identified in lamellar bodies**. Type II cells were cultured overnight on glass cover slips. The cells were fixed and subjected to double-labeling with antibodies against EHD1 or CAIV and LB-180 proteins. Shown are the representative images indicating the co-localization of EHD1 or CAIV with the LB-180 protein.

Based on the availability of antibodies, we selected several important proteins identified by trypsinolytic fingerprinting from each functional category and verified their identities by Western blotting and immunohistochemistry. Actin, Annexin A2, calreticulin, EH domain-containing 1 protein (EHD1), Rho-GDP dissociation inhibitor alpha (GDI-alpha) and vimentin were confirmed to be present in lung tissue and lamellar bodies as seen by Western blot (Fig. 4). Lamellar bodies contain more EHD1 than freshly isolated type II cell lysate. Only a small portion of GDI-alpha and actin were present in lamellar bodies, indicating these proteins were also localized elsewhere in type II cells. Annexin A2 and calreticulin existed in lamellar bodies and freshly isolated type II cell lysate in a similar amount. Vimentin was abundantly present in lamellar bodies, however undetectable in freshly isolated type II cells. The reason is unclear but could be due to the enrichment of this protein in lamellar bodies and the detection sensitivity. EHD1 along with the carbonic anhydrase IV (CAIV) protein were further investigated for their cellular localization in overnight-cultured type II cells because of their suitability for double-immunostaining. As seen in Fig. 5, both EHD1 and CAIV partially colocalized with the lamellar body marker, LB-180. The localization of these proteins at lamellar bodies further suggests their roles in lamellar body function.

**Figure 6:**
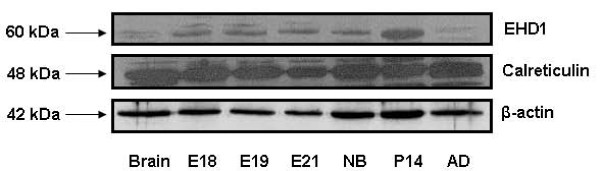
**Developmental profiles of EHD1 and calreticulin in the lung**. Tissues were collected from fetal lungs with gestational days 18, 19, and 21 (E18, E19, E21) and the lungs from newborn (NB), postnatal day 14 (P14) and adult (AD) rats. The protein levels of EHD1 and calreticulin were determined by Western blot. The β-actin was used as a loading control.

Since some of the proteins are developmentally regulated, we also determined the developmental profiles of two of the identified proteins (Fig. 6). The protein expression levels of EHD1 peaked postnatally. Calreticulin was expressed constantly throughout lung development.

## Discussion and conclusion

Although proteomic analyses have been performed on several secretory granules [7-9], proteomic prolifes of lamellar bodies of alveolar type II cells have remained a mystery. Here, we have identified 44 proteins present in lamellar bodies by using one- and two dimensional electrophoresis and trypsinolytic fingerprinting. The identification of these proteins provides a basis for further studying their functions in lamellar body exocytosis and biogenesis.

The proteins in lamellar bodies fell into diversified functional categories. Several calcium-binding proteins were identified, including the annexin family and calreticulin. There are some controversies as to which annexins are expressed in alveolar type II cells in literature. We previously showed that annexin A1, A2, A3, A6, but not A4 and A5 were present in rat alveolar type II cells as determined by immunoblotting [14]. However, Sohma et al. were able to detect annexin A4 in type II cell lysate [15]. Mayran et al. observed the presence of annexin A1, A4 and A6 in type II cells by using immunohistochemistry [16]. The inconsistency is likely due to differences among the antibodies used in the respective experiments. Using the proteomic approach, we identified seven members of the annexin family, annexin A1-7, in lamellar bodies.

Consistent with its representation in the lamellar body proteome, annexin A2 is important in various aspects of membrane trafficking [17]. A series of studies from our laboratory support a role of annexin A2 in lung surfactant secretion. It mediates the fusion between lamellar bodies and the plasma membrane [11,14]. The silencing of annexin A2 by RNA interference inhibits regulated lung surfactant secretion [18]. Annexin A2 functions in lung surfactant secretion via its interaction with SNAP-23 [13]. Disruption of lipid rafts, a platform organizing exocytotic proteins on a membrane, reduced annexin A2-mediated fusion of lamellar bodies with the plasma membrane [12]. In addition to annexin A2, annexin A7 has also been proposed to be involved in regulating lung surfactant secretion [19].

We find that annexin A1, A5 and A6 are the components of the lamellar body proteome. Annexin A1 and A6 have been reported to play roles in endocytosis [20,21]. Annexin A5 was reported to be secreted from type II cells. Because its secretion was stimulated by phorbol-12-myristate-13-acetate (PMA) and inhibited by SP-A, showing the same pattern as that of surfactant secretion [22], annexin A5 may be released through lamellar bodies. Annexins play important roles in governing lamellar body exocytosis and endocytosis, membrane organization, membrane cytoskeleton linkage and ion conductance.

Soluble N-ethylmaleimide-sensitive fusion protein attachment protein receptors (SNAREs) are a conserved mechanism for membrane targeting, docking and fusion [23]. We have previously shown that the plasma membrane SNAREs, syntaxin 2 and SNAP-23 and their co-factor, α-SNAP are essential for regulated surfactant secretion [24,25]. We have also detected the vesicle SNARE, VAMP-2 in lamellar bodies of type II cells using immunofluoresence [26]. However, our proteomic analysis of lamellar bodies did not show the presence of VAMP-2. The possible reasons include its low abundance or loss during sample preparation and separation.

We note that calreticulin is a component of the lamellar body proteome. Calreticulin is a calcium-binding protein for calcium storage in the endoplasmic reticulum (ER) [27]. It has also been reported to act as an important modulator of the regulation of gene transcription by nuclear hormone receptors [28]. Consistent with its representation in our demonstration of the lamellar body proteome, lamellar bodies contain a high level of calcium, especially those in the apical area. It was reported that the exocytotic lamellar bodies contain significantly higher calcium compared to those in the perinuclear area [29]. This suggests that this high content of calcium in lamellar bodies may be a supply for the increase in local calcium concentration during fusion events. Thus, our results indicate that calreticulin may be the main calcium-binding protein in lamellar bodies and may control the release of calcium ions during exocytosis.

In agreement with the nature of lamellar bodies as discrete cytological structures, we identified several cytoskeletal proteins in lamellar bodies by proteomic analysis. These proteins include β-actin, myosin-9, tropomysin 1, moesin, cytokeratin 19 and vimentin. Cytoskeleton is composed of 3 filamentous structures: actin filaments, intermediate filaments and microtubes of myosin. It has been reported that actin [30] and microtubules [31] play a role in the transportation of lamellar bodies. The cortical cytoskeleton in type II cells undergoes disassembly and assembly after being stimulated with lung surfactant secretagogues [32]. This process is regulated by annexin A2 and is essential for the lamellar body's access to the apical plasma membrane. Cytokertain 19 and vimentin are components of the intermediate filaments [33]. Tropomysin 1 is a ~40 kDa ubiquitous protein that is associated with the actin filaments and regulates the functions of actin cytoskeleton [34]. Moesin is a member of the ezrin/adixin/moesin family and plays a role in the linking of the plasma membrane and cytoskeleton [35]. These proteins may also be involved in the movement of lamellar bodies.

Among the major surfactant-related proteins described here, SP-A and SP-B are known components of lung surfactant. However, we did not detect SP-C. This is likely due to its loss during sample preparation and gel separation since SP-C is a hydrophobic and low molecular mass peptide. We also observed that several other proteins previously implicated in lamellar body biogenesis are in fact part of the lamellar body proteome. ATP-binding cassette transporter A3 (ABCA3) is a unique type II cell marker that was originally identified by a monoclonal antibody screen using lamellar body limited membrane [36]. It transports lipids into lamellar bodies [37]. ABCA3 mutations result in fatal neonatal lung diseases [38]. Peroxiredoxin-6, which possesses both phospholipase A_2 _and peroxidase activity, is responsible for the degradation of the main recycled surfactant phospholipid, Dipalmitoylphosphatidylcholine (DPPC), in type II cells [39]. Apolipoprotein A is the major apoprotein of high-density-lipoprotein (HDL) [40]. The identification of apolipoproteins in lamellar bodies suggests that the cholesterol of surfactant may originate from HDL. Our findings suggest that these proteins are important components of lamellar bodies, thus demanding closer examination of their roles in lamellar body structure, function and regulation.

Our results support the idea that lamellar bodies are lysosome-related organelles that retain some lysosome-like features. We note that several lysosome-like proteins are the components of lamellar bodies. Along with lysosome membrane protein II [41], some proteases (Dipeptidyl peptidase IV) [42] and protease inhibitors (Serpinh1 and alpha-1-antitrypsin precursor) [43,44] were identified, which suggests that lamellar bodies may not only store surfactant, but they may also play a role in surfactant processing. We also identified the vacuolar proton transporting ATPase (V-ATPase) subunit (H^+^-transporting two-sector ATPase alpha chain precursor). V-ATPases may pump protons into lamellar bodies to maintain the low pH inside lamellar bodies [4,45]., which is essential for surfactant processing [46,47] and calcium uptake [48].

Our analyses of the lamellar bodies also implicate novel proteins that may regulate lamellar body function. One is the EH domain-containing 1 protein (EHD1). The EH domain includes an EF-calcium-binding motif, a highly conserved ATP/GTP-binding domain and a central coiled-coil structure [49]. It has been reported that EHD1 interacts with SNAP-29 and plays a role in the endocytosis of Insulin-like Growth Factor 1 (IGF1) receptors [50]. Its role in the regulation of endocytic recycling has been further confirmed in other systems [51]. While EHD1 has not been previously implicated in endocytosis of surfactant, our findings imply that EHD1 may have a role in lamellar body function.

Our indentificatoin of GDI alpha as a major component of lamellar bodies suggest that it may be involved in the docking of lamellar bodies at the plasma membrane. Rab proteins are small GTP-binding proteins that play roles in vesicular trafficking of molecules between cellular organelles [52]. They serve as functional switches for the GTP-GDP exchange reaction. Rab GDP dissociation inhibitors (GDIs) can reduce the rate of GDP dissociation from Rab proteins [53]. Rab GDI alpha has been reported to bind Rab3A and modulates their activity and vesicle-mediated transport [54]. Small GTP-binding proteins are associated with lamellar bodies [55]. Rab3D only exists in subpopulations of lamellar bodies (~25% close to the apical membrane) [56].

Carbonic anhydrase (CA) is a zinc metalloenzyme, which reversibly catalyses the conversion of CO_2 _to HCO_3 _and a proton [57]. They are predominantly involved in maintaining acid-base balance. CAII was present in both lung type I and type II cells [58,59]. CAIV were detected in alveolar capillary endothelium but not on large blood vessels [60]. Our results indicated that CAIV was localized on the lamellar bodies of type II cells. Lamellar bodies maintain acidic interiors, which favor not only processing of SP-B and SP-C but also aggregation of surfactant lipids [46,47,61]. Thus, the presence of CAIV on lamellar bodies might be one of the additional mechanisms in maintaining and regulating the acidic interiors.

Our results document the first comprehensive proteome of the isolated rat lung lamellar bodies. However, lamellar bodies likely contain proteins that were not detected in our study due to their low abundance or due to individual molecular masses and isoelectric point values that hinder analysis by gel-based techniques. There may also be more proteins that we could not identify due to the incomplete nature of the *Rattus *sequence database. It is also noted that a few identified proteins have lower molecular masses than the calculated values. This is probably due to degradation or processing. Our initial characterization of the protein constituents of lamellar bodies provides a platform for further studying lamellar body biogenesis and surfactant secretion.

## Methods

### Reagents and chemicals

The ReadyPrep 2-D cleanup kit, ReadyPrep reduction-alkylation kit, ReadyPrep protein extraction kit, non-linear pH 3-10 ReadyStrip IPG strips and horse-radish peroxidase (HPR)-conjugated goat anti-rabbit and anti-mouse antibodies were from Bio-Rad Laboratories (Hercules, CA). Rabbit polyclonal anti-β-actin antibodies were from Sigma (St Louis, MO). Monoclonal anti-annexin A2 and rabbit polyclonal anti-Rho-GDP dissociation inhibitor (GDI)-alpha antibodies were from Invitrogen (Carlsbad, CA). Rabbit polyclonal anti-vimentin, anti-dipeptidyl peptidase IV (CD26) and anti-calreticulin antibodies were from Abcam (Cambridge, MA). Polyclonal rabbit anti-EH domain-containing 1 protein (EHD1) and anti-carbonic anhydrase IV (CAIV) antibodies were from Santz Cruz (Santz Cruz, CA). Mouse anti-LB-180 antibody was from Covance Research Products (Richmond, VA).

### Isolation of lamellar bodies from rat lung

Lamellar bodies were isolated from rat lungs by upward flotation on a discontinuous sucrose gradient, as described by Chander et al. [10] and Chattopadhyay et al. [11]. The Oklahoma State University Animal Use and Care Committee approved all animal procedures used in this study. A perfused rat lung was homogenized in 1 M sucrose and loaded at the bottom of a sucrose gradient (0.2, 0.3, 0.4, 0.5, 0.6, 0.7, and 0.8 M). After centrifugation at 80,000 × *g *for 3 hours, the lamellar body fraction was collected at the 0.4 and 0.5 M interface, and diluted to 0.24 M with cold water. Lamellar bodies were then spun down at 20,000 × *g *and resuspended in 0.24 M sucrose containing 10 mM Tris and 50 mM Hepes (pH 7.0). The protein concentration of lamellar bodies was determined by the RC-DC protein assay (Bio-Rad). We typically used 6 rats for the isolation of lamellar bodies, which yielded ~1 mg protein of lamellar bodies.

### One-dimensional SDS-PAGE

Lamellar bodies (~100 μg protein) were directly lysed in 1 × SDS sample buffer and fractionated on 10% SDS-PAGE under reducing conditions. The gel was subjected to colloidal coomassie brilliant blue staining to visualize protein bands as described below.

### Two-dimensional gel electrophoresis

Lamellar bodies (~500 μg protein) were processed by the ReadyPrep 2-D cleanup kit according to the manufacturer's protocol. The pellet was air-dried at room temperature and resuspended in an appropriate volume of 2-D rehydration/sample buffer (8 M Urea, 2% CHAPS, 50 mM DTT, 0.2% Bio-Lyte 3/10 ampolyte, and 0.002% bromophenol blue). The non-solubilized particulate matter was removed by brief centrifugation and the supernatant was stored at -80°C or used to passively hydrate 17 cm non-linear, pH 3–10, immobilized pH gradient strips (IPG) and isoelectric focusing was performed on a Protean IEF Cell (Bio-Rad). The IPG strips were equilibrated in equilibration buffers I (6 M urera, 0.375 M Tris, pH 8.8, 2% SDS, 20% glycerol. 2% (w/v) DTT) and II (6 M urera, 0.375 M Tris, pH 8.8, 2% SDS, 20% glycerol, 2.5% (w/v) iodoacetaminde) and loaded onto 8 – 16% gradient gel where the proteins were resolved in a secocd dimension gel electrophoresis. After SDS-PAGE, the gels were fixed with 40% ethanol and 10% acetic acid overnight, followed by washing with water twice for 10 min. The colloidal coomassie brilliant staining working solution was made by mixing 80% stock solution (0.1% coomassie brilliant blue G-250, 2% ortho-phosphoric acid, 10% ammonium sulfate) with 20% methanol. The gels were stained overnight and destained with 1% acetic acid until satisfactory destaining was achieved.

### MALDI-TOF mass spectrometry

Acrylamide bands or spots were harvested, washed with ammonium bicarbonate and digested for 8 h with 8 μg/ml trypsin. Trypsonolysis products were extracted and analyzed by reflectron MALDI-TOF mass spectrometry (Applied Biosystems DE-Pro) using alpha-cyano-4-hydroxy cinnamic acid as a matrix. Spectra were calibrated to 100 ppm mass accuracy using a mix of 5 known peptides (m/z 904 to 2465) spotted proximal to each sample on the MALDI probes.

### Protein identification analysis

Protein identification was carried out by searching the peptide spectra against the Mass Spectrometry protein sequence database (MSDB) using the Mascot web based search engine. The search parameters used were: taxonomy, *Rattus*; allow up to 1 missed cleavage; variable modifications, carbamidomethyl (C), oxidation (M), propionamide (C), and pyro-glu (N-term Q); mass value, MH+; allowed error, 100 ppm.

### Alveolar type II cell isolation

Type II cells were isolated from male Sprague-Dawley rats (180–200 g) as previously described [14]. Briefly, the perfused and lavaged lungs were digested with elastase (3 U/ml) at 37°C. The lung tissues were chopped and filtered through 160-, 37- and 15-μm mesh size nylon gauze. The cells were centrifuged, resuspended in culture medium and panned in a rat IgG-coated bacteriological plastic dish to remove alveolar macrophages. The unattached cells were collected. The resultant type II cells had a purity of >90% and a viability of >95%.

### Western blotting

Lung tissue, freshly isolated type II cells and lamellar bodies (20 μg protein each) were fractionated on 10% SDS-PAGE and transferred to a nitrocellulose membrane. The membrane was blocked with 5% fat-free milk in Tris-buffered-saline with Tween 20 (TTBS, 20 mM Tris-HCl, pH 7.6, 150 mM NaCl and 0.1% Tween 20). The membrane was then incubated with appropriate primary antibodies (β-Actin, 1:2000; Annexin A2, 1:1000; Calreticulin, 1:1000; EHD1, 1:200; GDP-1, 1 μg/ml; Vimentin, 1:500) at 4°C overnight, and then with secondary antibody (1:2500) at room temperature for 1 h. Finally, the signal was developed with ECL reagents and exposed to X-ray film.

### Immunocytochemistry

Type II cells were suspended in DMEM (supplemented with 10% FBS, non-essential amino acids and antibiotics) and cultured on glass cover slips overnight. Following culture, the cells were washed three times with ice-cold 50 mM phosphate buffered saline (PBS) and fixed in 4% paraformaldehyde at room temperature for 20 min. The cells were then permeablized with 0.5% Triton X-100 for 20 min at room temperature and then incubated with 10% fetal bovine serum to prevent non-specific binding of antibodies. The cells were subsequently incubated with polyclonal rabbit anti-EHD1 or anti-CAIV at 1:50 and mouse anti-LB-180 at 1:500 overnight at 4°C. After washing 3 times with PBS for 5 min each, the cells were incubated with alexa 488-conjugated goat anti-rabbit and Cy3-conjugated goat anti-mouse antibodies at a 1:250 dilution for 1 hour at room temperature. After washing 3 times with PBS, the cover slips were mounted onto glass slides. Anti-fade solution [60% (v/v) glycerol; 1.5% (w/v) n-propyl gallate in 50 mM PBS] was added to prevent fading before sealing the cover slips. Pictures were taken with a Nikon Eclipse E600 microscope (Nikon, Lewisville, TX). Negative controls were incubated with secondary antibodies only. Additional controls were included to eliminate the possibility of cross-reactivity among the antibodies (data not shown).

## Authors' contributions

PW performed proteomic analysis and Western blot and drafted the manuscript. NRC performed immunostaining. JN performed part of Western blot analysis. NRC and SA participated in 2-D gel. SH participated in MALDI-TOF MS. LL conceived of the study, participated in its design and coordination and helped to draft the manuscript. All authors read and approved the final manuscript.

## References

[B1] Hallman M, Glumoff V, Ramet M Surfactant in respiratory distress syndrome and lung injury. Comp Biochem Physiol A Mol Integr Physiol 2001 May ;129 (1 ):287 -94.

[B2] Wright JR, Clements JA (1987). Metabolism and turnover of lung surfactant. Am Rev Respir Dis.

[B3] Chander A, Fisher AB (1990). Regulation of lung surfactant secretion.. Am J Physiol.

[B4] Chander A, Johnson RG, Reicherter J, Fisher AB (1986). Lung lamellar bodies maintain an acidic internal pH. J Biol Chem.

[B5] Andreeva AV, Kutuzov MA, Voyno-Yasenetskaya TA (2007). Regulation of Surfactant Secretion in Alveolar Type II cells. Am J Physiol Lung Cell Mol Physiol.

[B6] Dietl P, Haller T (2005). Exocytosis of lung surfactant: from the secretory vesicle to the air-liquid interface. Annu Rev Physiol.

[B7] Brunner Y, Coute Y, Iezzi M, Foti M, Fukuda M, Hochstrasser D, Wollheim C, Sanchez JC (2007). Proteomic analysis of insulin secretory granules. Mol Cell Proteomics.

[B8] Chen X, Walker AK, Strahler JR, Simon ES, Tomanicek-Volk SL, Nelson BB, Hurley MC, Ernst SA, Williams JA, Andrews PC (2006). Organellar proteomics: analysis of pancreatic zymogen granule membranes. Mol Cell Proteomics.

[B9] Muth E, Driscoll WJ, Smalstig A, Goping G, Mueller GP (2004). Proteomic analysis of rat atrial secretory granules: a platform for testable hypotheses. Biochim Biophys Acta.

[B10] Chander A, Dodia CR, Gil J, Fisher AB (1983). Isolation of lamellar bodies from rat granular pneumocytes in primary culture. Biochim Biophys Acta.

[B11] Chattopadhyay S, Sun P, Wang P, Abonyo B, Cross NL, Liu L (2003). Fusion of lamellar body with plasma membrane is driven by the dual action of annexin II tetramer and arachidonic acid. J Biol Chem.

[B12] Chintagari NR, Jin N, Wang P, Narasaraju TA, Chen J, Liu L (2006). Effect of cholesterol depletion on exocytosis of alveolar type II cells. Am J Respir Cell Mol Biol.

[B13] Wang P, Chintagari NR, Gou D, Su L, Liu L (2007). Physical and Functional Interactions of SNAP-23 with Annexin A2. Am J Respir Cell Mol Biol.

[B14] Liu L, Wang M, Fisher AB, Zimmerman UJP (1996). Involvement of annexin II in exocytosis of lamellar bodies from alveolar epithelial type II cells. Am J Physiol.

[B15] Sohma H, Matsushima N, Watanabe T, Hattori A, Kuroki YX, Akino T (1995). Ca(2+)-dependent binding of annexin IV to surfactant protein A and lamellar bodies in alveolar type II cells. Biochem J.

[B16] Mayran N, Traverso V, Maroux S, Massey-Harroche D (1996). Cellular and subcellular localizations of annexins I, IV, and VI in lung epithelia. Am J Physiol.

[B17] Gerke V, Creutz CE, Moss SE (2005). Annexins: linking Ca2+ signalling to membrane dynamics. Nat Rev Mol Cell Biol.

[B18] Gou D, Wang P, Jin N, Liu L (2004). Silencing of annexin II in primary culture of alveolar epithelial type II cells. Annexins.

[B19] Chander A, Sen N, Spitzer AR (2001). Synexin and GTP increase surfactant secretion in permeabilized alveolar type II cells. Am J Physiol Lung Cell Mol Physiol.

[B20] Pons M, Grewal T, Rius E, Schnitgerhans T, Jackle S, Enrich C (2001). Evidence for the Involvement of annexin 6 in the trafficking between the endocytic compartment and lysosomes. Exp Cell Res.

[B21] Seemann J, Weber K, Osborn M, Parton RG, Gerke V (1996). The association of annexin I with early endosomes is regulated by Ca2+ and requires an intact N-terminal domain. Mol Biol Cell.

[B22] Sohma H, Ohkawa H, Akino T, Kuroki Y (2001). Binding of annexins to lung lamellar bodies and the PMA-stimulated secretion of annexin V from alveolar type II cells. J Biochem.

[B23] Chen YA, Scheller RH (2001). SNARE-mediated membrane fusion. Nat Rev Mol Cell Biol.

[B24] Abonyo BO, Wang P, Narasaraju TA, Rowan WH, McMillan DH, Zimmerman UJ, Liu L (2003). Characterization of alpha-Soluble N-Ethylmaleimide-Sensitive Fusion Attachment Protein in Alveolar Type II Cells: Implications in Lung Surfactant Secretion. Am J Respir Cell Mol Biol.

[B25] Abonyo BO, Gou D, Wang P, Narasaraju T, Wang Z, Liu L (2004). Syntaxin 2 and SNAP-23 are required for regulated surfactant secretion. Biochemistry.

[B26] Zimmerman UJ, Malek SK, Liu L, Li HL (2000). Proteolysis of synaptobrevin, syntaxin, and SNAP-25 in alveolar epithelial type II cells. IUBMB Life.

[B27] Michalak M, Milner RE, Burns K, Opas M (1992). Calreticulin. Biochem J.

[B28] Burns K, Duggan B, Atkinson EA, Famulski KS, Nemer M, Bleackley RC, Michalak M (1994). Modulation of gene expression by calreticulin binding to the glucocorticoid receptor. Nature.

[B29] Eckenhoff RG, Somlyo AP (1988). Rat lung type II cell and lamellar body: elemental composition in situ. Am J Physiol.

[B30] Tsilibary EC, Williams MC (1983). Actin and secretion of surfactant. J Histochem Cytochem.

[B31] Brown LA, Pasquale SM, Longmore WJ (1985). Role of microtubules in surfactant secretion. J Appl Physiol.

[B32] Singh TK, Abonyo B, Narasaraju TA, Liu L (2004). Reorganization of cytoskeleton during surfactant secretion in lung type II cells: a role of annexin II. Cell Signal.

[B33] Buccheri G, Ferrigno D (2001). Lung tumor markers of cytokeratin origin: an overview. Lung Cancer.

[B34] Gunning P, O'Neill G, Hardeman E (2008). Tropomyosin-based regulation of the actin cytoskeleton in time and space. Physiol Rev.

[B35] Lankes WT, Furthmayr H (1991). Moesin: a member of the protein 4.1-talin-ezrin family of proteins. Proc Natl Acad Sci U S A.

[B36] Zen K, Notarfrancesco K, Oorschot V, Slot JW, Fisher AB, Shuman H (1998). Generation and characterization of monoclonal antibodies to alveolar type II cell lamellar body membrane. Am J Physiol.

[B37] Cheong N, Madesh M, Gonzales LW, Zhao M, Yu K, Ballard PL, Shuman H (2006). Functional and trafficking defects in ATP binding cassette A3 mutants associated with respiratory distress syndrome. J Biol Chem.

[B38] Bullard JE, Wert SE, Nogee LM (2006). ABCA3 deficiency: neonatal respiratory failure and interstitial lung disease. Semin Perinatol.

[B39] Wu YZ, Manevich Y, Baldwin JL, Dodia C, Yu K, Feinstein SI, Fisher AB (2006). Interaction of surfactant protein A with peroxiredoxin 6 regulates phospholipase A2 activity. J Biol Chem.

[B40] Davidson WS, Thompson TB (2007). The structure of apolipoprotein A-I in high density lipoproteins. J Biol Chem.

[B41] Salaun B, de Saint-Vis B, Pacheco N, Pacheco Y, Riesler A, Isaac S, Leroux C, Clair-Moninot V, Pin JJ, Griffith J, Treilleux I, Goddard S, Davoust J, Kleijmeer M, Lebecque S (2004). CD208/dendritic cell-lysosomal associated membrane protein is a marker of normal and transformed type II pneumocytes. Am J Pathol.

[B42] Hildebrandt M, Reutter W, Gitlin JD (1991). Tissue-specific regulation of dipeptidyl peptidase IV expression during development. Biochem J.

[B43] Wang H, Sammel MD, Tromp G, Gotsch F, Halder I, Shriver MD, Romero R, Strauss JF (2008). A 12-bp deletion in the 5'-flanking region of the SERPINH1 gene affects promoter activity and protects against preterm premature rupture of membranes in African Americans. Hum Mutat.

[B44] Venembre P, Boutten A, Seta N, Dehoux MS, Crestani B, Aubier M, Durand G (1994). Secretion of alpha 1-antitrypsin by alveolar epithelial cells. FEBS Lett.

[B45] Wadsworth SJ, Spitzer AR, Chander A (1997). Ionic regulation of proton chemical (pH) and electrical gradients in lung lamellar bodies. Am J Physiol.

[B46] Beers MF (1996). Inhibition of cellular processing of surfactant protein C by drugs affecting intracellular pH gradients. J Biol Chem.

[B47] Chander A, Sen N, Wu AM, Higgins S, Wadsworth S, Spitzer AR (1996). Methylamine decreases trafficking and packaging of newly synthesized phosphatidylcholine in lamellar bodies in alveolar type II cells. Biochem J.

[B48] Wadsworth SJ, Chander A (2000). H+-and K+-dependence of Ca2+ uptake in lung lamellar bodies. J Membr Biol.

[B49] Mintz L, Galperin E, Pasmanik-Chor M, Tulzinsky S, Bromberg Y, Kozak CA, Joyner A, Fein A, Horowitz M (1999). EHD1--an EH-domain-containing protein with a specific expression pattern. Genomics.

[B50] Rotem-Yehudar R, Galperin E, Horowitz M (2001). Association of insulin-like growth factor 1 receptor with EHD1 and SNAP29. J Biol Chem.

[B51] Naslavsky N, Boehm M, Backlund PS, Caplan S (2004). Rabenosyn-5 and EHD1 interact and sequentially regulate protein recycling to the plasma membrane. Mol Biol Cell.

[B52] Bock JB, Matern HT, Peden AA, Scheller RH (2001). A genomic perspective on membrane compartment organization. Nature.

[B53] Bachner D, Sedlacek Z, Korn B, Hameister H, Poustka A (1995). Expression patterns of two human genes coding for different rab GDP-dissociation inhibitors (GDIs), extremely conserved proteins involved in cellular transport. Hum Mol Genet.

[B54] D'Adamo P, Menegon A, Lo NC, Grasso M, Gulisano M, Tamanini F, Bienvenu T, Gedeon AK, Oostra B, Wu SK, Tandon A, Valtorta F, Balch WE, Chelly J, Toniolo D (1998). Mutations in GDI1 are responsible for X-linked non-specific mental retardation. Nat Genet.

[B55] Rubins JB, Panchenko M, Shannon TM, Dickey BF (1992). Identification of ras and ras-related low-molecular-mass GTP-binding proteins associated with rat lung lamellar bodies. Am J Respir Cell Mol Biol.

[B56] van WL, de Graaff AM, Jamieson JD, Batenburg JJ, Valentijn JA (2004). Rab3D and actin reveal distinct lamellar body subpopulations in alveolar epithelial type II cells. Am J Respir Cell Mol Biol.

[B57] Purkerson JM, Schwartz GJ (2007). The role of carbonic anhydrases in renal physiology. Kidney Int.

[B58] Chen J, Lecuona E, Briva A, Welch LC, Sznajder JI (2008). Carbonic anhydrase II and alveolar fluid reabsorption during hypercapnia. Am J Respir Cell Mol Biol.

[B59] Fleming RE, Moxley MA, Waheed A, Crouch EC, Sly WS, Longmore WJ (1994). Carbonic anhydrase II expression in rat type II pneumocytes. Am J Respir Cell Mol Biol.

[B60] Fleming RE, Crouch EC, Ruzicka CA, Sly WS (1993). Pulmonary carbonic anhydrase IV: developmental regulation and cell-specific expression in the capillary endothelium. Am J Physiol.

[B61] Voorhout WF, Veenendaal T, Haagsman HP, Weaver TE, Whitsett JA, van Golde LM, Geuze HJ (1992). Intracellular processing of pulmonary surfactant protein B in an endosomal/lysosomal compartment. Am J Physiol.

